# Sodium Toxicity: Should NaOH Be Substituted by KOH in Plant Tissue Culture?

**DOI:** 10.3389/fpls.2022.829768

**Published:** 2022-02-04

**Authors:** Oumar Doungous, Jameel M. Al-Khayri, Modeste Kan Kouassi

**Affiliations:** ^1^The Central and West African Virus Epidemiology (WAVE), Biotechnology Laboratory, Ekona Regional Research Centre, Institute of Agricultural Research for Development, Yaoundé, Cameroon; ^2^Department of Agricultural Biotechnology, College of Agriculture and Food Sciences, King Faisal University, Al-Ahsa, Saudi Arabia; ^3^The Central and West African Virus Epidemiology (WAVE), Plant Tissue Culture Laboratory, Pôle Scientifique et d'Innovation de Bingerville, Université Félix Houphouët-Boigny, Abidjan, Côte d'Ivoire

**Keywords:** NaOH toxicity, KOH, medium pH adjustment, dissolution of growth regulators, nutrient imbalance

## Introduction

Plant tissue culture is an attractive system that involves growing cells, tissues and organs of plants on artificial media under a controlled environment, with applications in food production through crop improvement and plant conservation. Plant tissue culture is also an important tool for the continuous production of active compounds including secondary metabolites and engineered molecules (Espinosa-Leal et al., 2018). Many tissue culture techniques exist and are used in research institutions as well as in commercial laboratories. One of the most important factors in plant tissue culture is the medium which is a defined formulation of inorganic salts and organic compounds providing nutrients to the cultured explants (George and de Klerk, 2008). The optimum nutrient concentration is a critical determinant for the growth and morphogenesis of tissues and the accumulation of secondary metabolites (Murthy et al., 2014).

Different culture media varying in the composition and level of macronutrients and micronutrients were developed, among which Murashige and Skoog medium or modifications from this formulation are the widely used for plant tissue culture (Murashige and Skoog, 1962; Phillips and Garda, 2019). Plant tissue culture media also contain some or all of the following components: vitamins, amino acids or nitrogen supplements, carbon source, undefined natural organic supplements, growth regulators and solidifying agents. Therefore, knowledge of the nutritional requirements of cultured cells, tissues and organs is important for choosing the most appropriate culture medium for the explant used and the targeted plant species, cultivar and genotype (Al-Khayri, 2011; Orlowska et al., 2021).

Most of *in vitro* culture studies were based on the types and concentrations of growth regulators which play a key role in determining the development pathway of plant cells and tissues (Amiri and Mohammadi, 2021; Mitrofanova et al., 2021). Considering that cells sensitivity to plant growth regulators may be affected by the mineral composition, studies across a wide range of species and applications have focused on the selection or the manipulation of basal salt composition for the optimization of the nutrient component for a particular response (Ramage and Williams, 2002; Niedz and Evens, 2007; Akin et al., 2017; Silva et al., 2019; Elyazid et al., 2021; Raji and Siril, 2021). Due to the diverse nutrition requirements of specific species, genotypes, developmental steps and the many interactions of the chemical nutrients, a number of approaches were developed for the estimation of the correct concentration balance of mineral nutrients and the optimization or customization of the medium. These approaches range from factorial or triangular method and response surface methodology to recent machine learning techniques (Khvatkov et al., 2019; Hameg et al., 2020; Hesami and Jones, 2020; Kabylbekova et al., 2020).

Another important factor for plant tissue culture is medium pH. It has a remarkable effect on the development of explants. It regulates the equilibrium at the cellular membrane level and controls the solubility and absorption of ions. Moreover, pH plays an important role in the solidification of the medium. As a rule of thumb, initial medium pH is adjusted to the value between 5.5–6.0 (Thorpe et al., 2008). An adjustment to the optimal value of the pH has to be done for almost all the media with alkali (NaOH or KOH) if the pH is low, or with acid (HCl) in case of high pH.

NaOH is also used for the preparation of stock solution of growth regulators in plant tissue culture. The toxicity of Na has been proven *in vivo* and *in vitro* in many studies, and most basic culture media are deprived from it. However, the negative effects of Na on mineral nutrition, morphogenesis and differentiation are largely unknown. In addition, no study has compared the effects of NaOH and KOH in plant tissue culture. Here we address the hypothesis that NaOH induces ion toxicity and nutritional imbalance. We argue that adjusting the pH of the medium and dissolving growth regulators with KOH instead of NaOH may be more beneficial for plant cell, tissue, and organ culture.

## Sodium In Tissue Culture Medium

Based on ecological considerations for survival and reproduction, Na is defined as a non-essential element for higher plants but has been shown to be essential for certain C4 plant species (Kronzucker et al., 2013; Krishnasamy et al., 2014). Most macronutrient formulations do not contain Na at all (Table 1). Only small amounts are incorporated into the media from the micronutrient components (Poothong et al., 2018). Even though Na is not considered as a major element, it can be added to the medium from other sources such as gelling agents used for medium solidification, or NaOH used for medium pH adjustment and for preparation of stock solution of growth regulators.

**Table 1:** Components and concentrations of Na and K in commonly used plant tissue culture basal media.

**Basal medium**	**MS**	**B5**	**WPM**	**DKW**	**SH**	**KC**	**VW**
**Macronutrient components of K and Na (mg L^−1^)**							
KNO_3_	1,900	2,500	–	–	2,500	–	525
K_2_SO_4_	–	–	990	1,559	-	–	–
KH_2_PO_4_	170	–	170	265	-	250	250
KCl	–	–	–	–	-	250	–
NaH_2_PO_4_·H_2_O	–	150	–	–	–	–	–
**Micronutrient components of K and Na (mg L^−1^)**							
KI	0.83	0.75	–	–	1	–	–
Na_2_MoO_4_·2H_2_O	0.25	0.25	0.25	0.39	0.1	–	–
Na_2_EDTA	37.3	37.3	37.3	45.4	20	–	–
**Molar concentration (mM)**							
K^+^	20.04	24.72	12.61	19.84	25.23	5.19	7.03
Na^+^	0.20	1.45	0.20	0.24	0.11	0	0

*Basal media were selected according to Phillips and Garda (2019). MS, Murashige and Skoog; B5, Gamborg; WPM, Woody Plant Medium; DKW, Driver and Kuniyuki Woody; SH, Schenk and Hildebrandt; KC, Knudson C Orchid; VW, Vacin and Went*.

NaOH is one of the solvents used for the preparation of stock solution of cytokinins and auxins (naphthalene acetic acid, 2,4 dichlorophenoxy acetic acid, and indol acetic acid). Generally, vitamins, amino acids, auxins, gibberellins and abscisic acid render the culture medium more acidic. Moreover, ascorbic acid or citric acid added to many media to control oxidative browning (Salazar-Vega et al., 2022) also lower the medium pH. Therefore, a pH adjustment to 5.5–6.0 with an alkali solution is needed. In most cases, this adjustment is done by adding NaOH to the medium (Shi et al., 2017). The lower the medium pH value, the higher the concentration of NaOH needed to increase the pH. Consequently, the use of NaOH either for dissolution of growth regulators or for pH adjustment increases the optimal Na^+^ concentrations initially defined for the medium. For apple tissue culture, Shi et al. (2017) demonstrated that adjustment of pH from 5.1 (initial) to 6.0 by adding NaOH leads to the significant increase in Na^+^ concentration. Furthermore, the Na may not be depleted easily from the culture medium and higher concentrations and activities of Na^+^ can cause salt stress.

## Salt Stress

Na^+^ is not required for many physiological and biochemical processes (Isayenkov and Maathuis, 2019). It is required only at the micronutrient level even for halophytes where Na is clearly beneficial if not essential (Krishnasamy et al., 2014; Yuan et al., 2019). Although under soil conditions low levels of Na^+^ can stimulate plant growth or other functions especially under K^+^ deficiency (Maathuis, 2014; Ferreira et al., 2020), this situation could not be applied in plant tissue culture due to the presence of potassium as macroelement in culture media (Table 1).

Salt stress reduces water uptake, leading to cell dehydration and changes in cell turgor, inducing osmotic stress. In the long term, accumulation of Na to toxic levels induces ionic toxicity leading to Ca^2+^, Mg^2+^ and K^+^ deficiency and to other nutrient imbalances (Munns and Tester, 2008; Hamani et al., 2021). Salt stress impairs growth both at whole plant and cellular level by low osmotic potential, nutritional imbalance, specific ion effect, or a combination of these factors.

In plant tissue culture, NaOH is not considered among the factors affecting the availability of nutrients and components of the medium. Although addition of NaOH for pH adjustment leads to the decrease in Mg^2+^ and Ca^2+^ concentration in the apple culture medium due to precipitation (Shi et al., 2017). Thus, *in vitro* cultures may be adversely affected by NaOH-induced nutritional disorders since it has been demonstrated that the concentrations and ratio of certain nutrients can affect the sensitivity of cells to growth regulators and other component of the medium as well as morphogenesis and differentiation (Leifert et al., 1995; Oberschelp and Goncalves, 2018). Nutrient deficiency or imbalance can cause abnormal physiological responses in plant cultures such as hyperhydricity, callus formation and necrosis (Reed et al., 2013; Teixeira da Silva et al., 2020). Additionally, plant cells and tissues performance may also be adversely affected by the alteration of metabolic pathways and osmotic adjustments. This is particularly important for protoplasts which are difficult to culture and are particularly useful to address essential biological questions regarding salt stress response (Gilliard et al., 2021).

The responses of plants to assess stress tolerance were commonly proven using *in vitro* stress assays. Most of these studies exposed plants to very high stress levels and scored very pronounced phenotypes such as germination rate, seedling survival, the development of visual symptoms such as bleaching or the induction of established stress markers (Skirycz et al., 2010; Claeys et al., 2014). Moreover, Na^+^ toxicity was tested with the interference of Cl^−^ toxicity, thus producing equivocal results (Genc et al., 2016). However, studies on the effect of salt stress in plant tissue culture have not received much attention and perhaps by overlooking or not considering Na^+^ from NaOH. This Na^+^ is not negligible and is likely to induce stress due to the fact that plant responses to salinity are complex and many reports confirmed that low stress levels can severely limit shoot growth without leading to other visible stress phenotypes (Claeys et al., 2014). Therefore, a better understanding of Na requirements of cultured cells and tissues, the role of Na on altered patterns of mineral nutrition, morphogenesis and differentiation of plant tissues is needed.

## The Choice Of KOH In Replacing NaOH

Studies on alkali might detect the negative effects of NaOH, improve media composition and *in vitro* protocols and contribute to the selection of most appropriate alkali solution for medium pH adjustment and dissolution of growth regulators. We can anticipate that adjusting medium pH and dissolving growth regulators with KOH instead of NaOH may be more beneficial for plant tissue culture, especially glycophytes, which include the vast majority of crop plants.

The choice of KOH in this note is due to multiple important attributes of this product. KOH is an alkali that is also used for medium pH adjustment in plant tissue culture (Elyazid et al., 2021). It can also be used for dissolution of growth regulators. There are chemical and structural similarities between Na^+^ and K^+^ in hydrated form (Isayenkov and Maathuis, 2019), thereby KOH can fulfill biophysical, physiological and biochemical processes achieved by NaOH. K^+^ is the only monovalent cation in plant cells which is essential for most higher plants and important for many enzymatic reactions, ionic and pH homeostasis, and maintenance of adequate membrane potential (Ahmad and Maathuis, 2014; Assaha et al., 2017). Potassium is among the chemicals whose increments have a positive effect on plant quality and growth parameters such as length or number of shoots (Poothong and Reed, 2015; Hunková et al., 2020). KOH has other advantages in that when higher concentrations are added to the medium, it is possible to adjust the additional amounts from multiple macronutrient components of potassium (Table 1). The average concentration of potassium of 13.6 mM was noticed in almost all culture media and the most common value (median) is 10.5 mM (George and de Klerk, 2008).

## Concluding Remarks And Future Perspectives

NaOH is commonly used for medium pH adjustment and for preparation of stock solution of growth regulators in plant tissue culture. As the use of NaOH will increase the initial concentration of Na^+^ from 0 or micromolar level to millimolar level in the culture medium, it is important to analyze the physiological, biochemical and molecular aspects of Na^+^, K^+^ and other ions uptake, sequestration, and transport in different protocols of *in vitro* cultures. An assessment of the effects of NaOH on nutrient availability, media optimization and development of *in vitro* cultures of different tissues/organs/genotypes is needed. Further approaches for the estimation of the correct concentration balance of mineral nutrients, modeling and the optimization of the medium should also take into consideration the Na derived from NaOH. As many studies reported the importance of potassium in managing the alkalinity problem, this assessment must also take into account KOH in view of selecting the appropriate alkali for each specific *in vitro* tissue culture. Since less is known on the relationship between mineral uptake and morphogenesis, we hope that this opinion article will also stimulate more investigations into the relationship between mineral uptake, transport, metabolism, and morphogenesis. Lastly, in addition to the commonly known soil salinity concept that is a major thread to agriculture, we believe that this communication can open a new concept of abiotic stresses affecting morphogenesis and differentiation of *in vitro*-grown plant cells, tissues and organs.

## Author Contributions

The manuscript was prepared by OD and reviewed by JA-K and MK. All authors contributed to the article and approved the submitted version.

## Funding

This work was supported, in whole or in part, by the Bill & Melinda Gates Foundation and The United Kingdom Foreign, Commonwealth & Development Office (FCDO) under Grant Number OPP1212988/INV-002969 to the Central and West African Virus Epidemiology (WAVE) Program for root and tuber crops - through a subgrant from Université Félix Houphouët-Boigny (UFHB) to the Institute of Agricultural Research for Development (IRAD). Under the grant conditions of the Foundation, a Creative Commons Attribution 4.0 Generic License has already been assigned to the Author Accepted Manuscript that might arise from this submission.

## Conflict of Interest

The authors declare that the research was conducted in the absence of any commercial or financial relationships that could be construed as a potential conflict of interest.

## Publisher's Note

All claims expressed in this article are solely those of the authors and do not necessarily represent those of their affiliated organizations, or those of the publisher, the editors and the reviewers. Any product that may be evaluated in this article, or claim that may be made by its manufacturer, is not guaranteed or endorsed by the publisher.
